# Elevated Hydrostatic Pressure Causes Retinal Degeneration Through Upregulating Lipocalin-2

**DOI:** 10.3389/fcell.2021.664327

**Published:** 2021-05-31

**Authors:** Azusa Yoneshige, Man Hagiyama, Yasutoshi Takashima, Satoru Ueno, Takao Inoue, Ryuichiro Kimura, Yoshiki Koriyama, Akihiko Ito

**Affiliations:** ^1^Department of Pathology, Faculty of Medicine, Kindai University, Osaka, Japan; ^2^Department of Ophthalmology, Faculty of Medicine, Kindai University, Osaka, Japan; ^3^Graduate School and Faculty of Pharmaceutical Sciences, Suzuka University of Medical Science, Suzuka, Japan

**Keywords:** glaucoma, intraocular pressure, retinal ganglion cells, apoptosis, gliosis, iron chelator

## Abstract

Elevation of intraocular pressure is a major risk factor for glaucoma development, which causes the loss of retinal ganglion cells (RGCs). Lipocalin 2 (Lcn2) is upregulated in glaucomatous retinae; however, whether Lcn2 is directly involved in glaucoma is debated. In this study, retinal explant cultures were subjected to increased water pressure using a two-chamber culture device, and Lcn2 protein levels were examined by immunoblotting. *In situ* TdT-mediated dUTP nick and labeling (TUNEL) and glial fibrillary acidic protein (GFAP) immunohistochemical assays were performed to assess apoptosis and gliosis, respectively. The neurotoxicity of Lcn2 in the retinal explant culture was determined with exogenous administration of recombinant Lcn2. The Lcn2 protein levels, percentage of TUNEL-positive cells, and GFAP-positive area were significantly higher in retinae cultured under 50 cm H_2_O pressure loads compared to those cultured under 20 cm H_2_O. We found that Lcn2 exhibited neurotoxicity in retinae at dose of 1 μg/ml. The negative effects of increased hydrostatic pressure were attenuated by the iron chelator deferoxamine. This is the first report demonstrating the direct upregulation of Lcn2 by elevating hydrostatic pressure. Modulating Lcn2 and iron levels may be a promising therapeutic approach for retinal degeneration.

## Introduction

Ocular hypertension is a major risk factor of glaucoma, which is characterized by the progressive loss of retinal ganglion cells (RGCs), resulting in irreversible vision loss. Although the reduction of intraocular pressure (IOP) is the only available treatment for glaucoma, the direct linkage between IOP elevation and retinal degeneration is yet to be found (Jayanetti et al., 2020; Pang, 2021).

Rodent models of glaucoma with ocular hypertension have provided valuable insights into the progression of retinal degeneration and potential treatments to provide neuroprotection against elevated IOP (Johnson and Tomarev, 2010; Struebing and Geisert, 2015). In the DBA/2J glaucoma mouse model, IOP elevation (5–8 mmHg higher than control mice) occurs by 9 months of age, caused by the blockage of aqueous outflow (Chang et al., 1999; Anderson et al., 2002; Libby et al., 2005). Another mouse model of glaucoma, which carries a mutation in the myocilin gene of the eye drainage structure, develops moderately elevated IOP levels (2–4 mmHg higher than control mice) after 1 year of age (Senatorov et al., 2006; Zhou et al., 2008). Both mouse models described exhibit a progressive reduction in RGCs, similar to human glaucoma. Furthermore, there are several experimental techniques available to mechanically induce ocular hypertension, including laser photocoagulation, episcleral venous cauterization, and anterior chamber injection; these techniques induce ocular hypertension rapidly compared to genetic models (Johnson and Tomarev, 2010). However, *in vivo* studies involving induced ocular hypertension are often complicated by additional factors, such as intraocular inflammation, hyphema, corneal opacity, IOP variability, ischemia, and aging (Rudzinski and Saragovi, 2005; Ishikawa et al., 2015).

Lipocalin 2 (Lcn2), also known as neutrophil gelatinase-associated lipocalin or 24p3, is a secreted protein that has diverse functions including immune regulation, iron transport, cell proliferation, cell differentiation, and cell death (Ferreira et al., 2015). Recent studies have discovered an association between Lcn2 expression and neurodegenerative diseases. The exogenous administration of amyloid beta stimulates Lcn2 production in primary astrocytes and Lcn2 enhances the sensitivity of primary neurons toward amyloid beta toxicity (Naude et al., 2012; Mesquita et al., 2014). Furthermore, clinical studies have revealed that Lcn2 levels in plasma and cerebrospinal fluid can be used as biomarkers for Alzheimer’s disease and other neurodegenerative dementias (Eruysal et al., 2019; Llorens et al., 2020). Lcn2 is also upregulated in the brain of patients affected by Alzheimer’s disease (Dekens et al., 2017), Parkinson’s disease (Kim et al., 2016), and multiple sclerosis (Al Nimer et al., 2016). Several reports of whole transcriptome analyses in rodent glaucoma models have revealed that *Lcn2* is upregulated in glaucomatous retinae (Fischer et al., 2004; Steele et al., 2006; Yang et al., 2007; Panagis et al., 2010; Guo et al., 2011; Yasuda et al., 2016; Ueno et al., 2018). We have also shown that *Lcn2* is remarkably upregulated soon after optic nerve injury, leading us to propose Lcn2 as a cause of RGC loss (Ueno et al., 2018). However, to our knowledge, the direct involvement of Lcn2 in glaucoma caused by ocular hypertension has not been investigated.

In a previous study, we devised a novel two-chamber culture system to investigate the mechanisms by which elevated intraluminal pressure causes cell or tissue degeneration. This system enabled us to load small amounts of water pressure (2–50 cm H_2_O) onto cells without disturbing standard culture conditions such as pH, oxygen, and carbon dioxide levels. We found that mouse primary neurons degenerated when the water pressure was above 30 cm H_2_O pressure load and that synaptic cell adhesion molecule 1 was involved in this process (Yoneshige et al., 2017). Additionally, we discovered that several columnar epithelial cell lines were growth-suppressed in a pressure-dependent manner and that the Hippo pathway and cytoskeleton dynamics were closely associated with this process (Hagiyama et al., 2017). In the present study, we investigated the involvement of Lcn2 in retinal degeneration induced by elevated hydrostatic pressure using our two-chamber culture system.

## Materials and Methods

### Mice

C57BL/6J and DBA/2J mice were obtained from CLEA Japan Inc. (Tokyo, Japan). IOP of DBA/2J mice was measured using a rebound tonometer (TONOLAB; Icare Finland Oy, Vantaa, Finland) according to the manufacturer’s instructions. IOP measurements were performed for four times every week at 3, 6, 9, and 12 months of age. All animal research was conducted in accordance with the Ethical Guidelines of Japan for the use of Animals in Research and was approved by the Committee for Animal Experiments of Kindai University (protocol code KAME-29-001).

### Retinal Organotypic Culture

The eyes of C57BL/6J mice were enucleated at 6–16 months of age and retinae were submerged in cold Dulbecco’s modified Eagle’s medium (DMEM). The retinae were placed at the bottom of a culture insert (transparent PET membrane with pore size 1.0 um; Corning, NC, United States) coated with Matrigel (Corning). Four retinae from two mice were cultured in a single culture insert with Neuronal culture medium (FUJIFILM Wako Pure Chemical Corp., Osaka, Japan) containing 50 ng/ml nerve growth factor (NGF; Millipore, CA, United States) and cultured within the two-chamber culture system for pressure loading. For neurotoxicity assay, one retina was cultured per well of 12-well culture plate (Corning) in Neuronal Culture Medium containing 1% FBS, 50 ng/ml NGF, and various concentrations of recombinant mouse Lcn2 (R&D systems, MN, United States) with or without 100 μM deferoxamine (Cayman Chemical, MI, United States). The next day, the retinae were fixed with paraformaldehyde and subjected to TUNEL assessment.

### Two-Chamber Culture System for Water Pressure Loading

**FIGURE 1:**
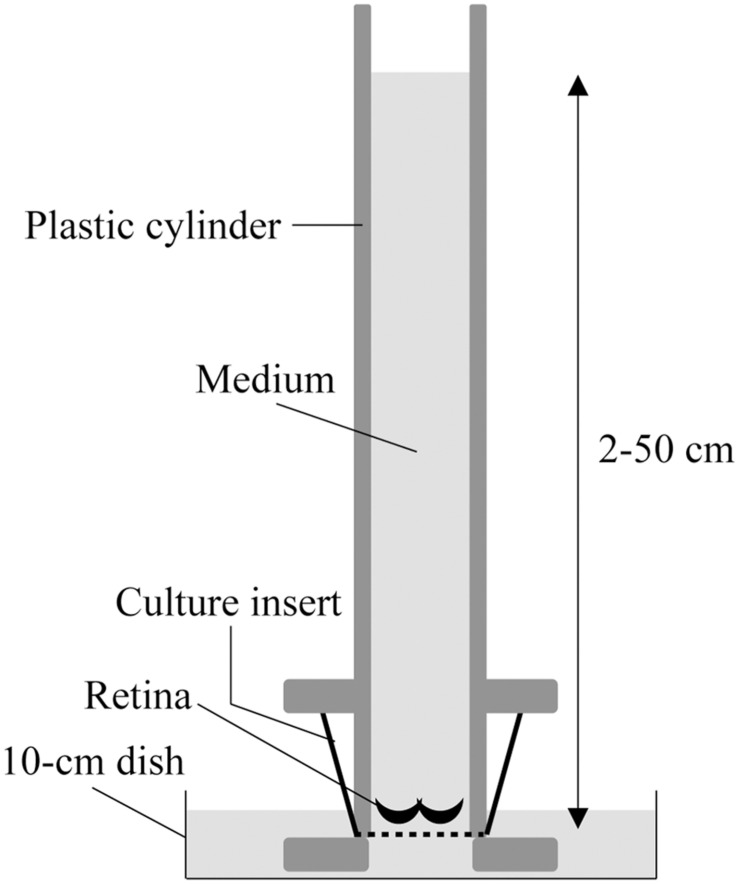
Schematic illustration of the two-chamber culture system for pressure loading. The retinae were submerged in media and placed at the bottom of a culture insert with Matrigel. The culture insert was connected to the bottom of a 50-cm-long plastic cylinder that enabled 2–50 cm H_2_O pressure loading, and was placed in a 10-cm culture dish where the culture insert made contact with the medium under free gas exchange conditions.

The two-chamber culture system was set up as previously described (Yoneshige et al., 2017). Briefly, the culture insert was placed between two chambers; the upper chamber was a 50-cm-long plastic cylinder that enabled 2–50 cm H_2_O pressure loading, and the lower chamber consisted of a 10-cm culture dish, in which the bottom of the culture insert made contact with the medium under free gas exchange conditions (Figure 1). The upper and lower chambers were filled with DMEM containing 10% fetal bovine serum (FBS) and 1% N2 supplement (Thermo Fisher Scientific, MA, United States). The entire two-chamber culture system was placed in a common CO_2_ incubator and maintained at 37°C with 5% CO_2_.

### Immunoblotting and Histological Examination

Polyclonal goat anti-Lcn2 (AF1857; R&D systems), monoclonal mouse anti-neuron specific enolase (NSE; BBS/NC/VI-H14; Dako, Glostrup, Denmark), monoclonal mouse anti-glial fibrillary acidic protein (GFAP; G-A-5; Sigma-Aldrich) and monoclonal mouse anti-glyceraldehyde-3-phosphate dehydrogenase (G3pdh; Medical and Biological Laboratories, Aichi, Japan) antibodies were the primary antibodies used in this study. Peroxidase-conjugated antibodies (GE Healthcare, IL, United States) or fluorescent-conjugated antibodies (Jackson ImmunoResearch, PA, United States) were used as secondary antibodies for immunoblotting or immunohistochemistry, respectively. Immunoblotting, TdT-mediated dUTP nick and labeling (TUNEL) assay, and immunohistochemical analysis of Lcn2 were carried out as described previously (Ueno et al., 2018). For the immunohistochemical analysis of GFAP, paraformaldehyde-fixed retinae were immersed in increasing concentrations of sucrose (10–30%) and embedded in frozen medium; cryosections (5 μm thickness) were further treated with 0.1% Triton X-100. TUNEL positivity, GFAP immunoreactivity, and nuclear counterstaining with 4′,6-diamidino-2-phenylindole (DAPI; Dojindo Laboratories, Kumamoto, Japan) were visualized with a BZ-X710 microscope (Keyence, Osaka, Japan) and measured using the BZ-X analysis software (Keyence).

### Laser-Captured Microdissection and Real-Time RT-PCR

The isolation of ganglion cell layer (GCL) from DBA/2J mouse retinae by laser-captured microdissection and the analysis of *Lcn2* mRNA levels were performed as previously described (Ueno et al., 2018). Briefly, the retinae of DBA/2J mice were immediately embedded in frozen embedding medium (SCEM-L1; Leica, Wetzlar, Germany) and 15-μm sections were mounted on membrane slides (2 μm-PEN-membrane; Leica), which were fixed with cold 5% acetic acid in ethanol. After staining with 0.025% toluidine blue solution, the GCL and inner plexiform layer (IPL) were dissected using a laser-captured microdissection system (LMD7000; Leica) and were directly captured in tissue lysis buffer. Total RNA was purified using the RNeasy Plus Micro kit (Qiagen, Hilden, Germany) and cDNA was synthesized using Superscript IV Reverse Transcriptase (Thermo Fisher Scientific) according to the manufacturers’ instructions. Expression levels of *Lcn2* in the GCL were determined by quantitative PCR using the StepOnePlus real-time PCR system (StepOne software v2.3 and Power SYBR Green PCR Master Mix; Applied Biosystems, CA, United States), using G3pdh as an internal control.

### Experimental Design and Statistical Analysis

Eight (20 cm and 50 cm + DFO) or ten retinae (2 and 50 cm) were used in organotypic culture for pressure loading, and twelve retinae were used for each treatment in neurotoxicity assay. Eight (6 and 12 months) or sixteen retinae (3 and 9 months) were used in DBA/2J mice analysis, and five retinae were used in C57BL/6J mice analysis. Each experiment was repeated at least 3 times and summary values were shown as mean ± SE. Significant differences among three or four groups were analyzed using the Steel-Dwass test. *P*-value ≤ 0.05 were considered to indicate statistical significance. Correlations were analyzed using Spearman’s rank test and considered significant if *p*-value ≤ 0.05 and *R*^2^ ≥ 0.1.

## Results

### Lcn2 Protein in the Retina Was Upregulated in Response to Elevated Loading Pressure in Our Two-Chamber Culture System, Which Was Mitigated by the Addition of Deferoxamine

To investigate retinal responses to IOP elevation, we developed a two-chamber culture system, which can load 2–50 cm H_2_O water pressure onto organotypic retinal explant cultures (Figure 1, Yoneshige et al., 2017). In adult mouse retinal explant cultures at atmospheric pressure (2 cm H_2_O), apoptotic RGCs were detected at 3 days *in vitro* (DIV) (Alarautalahti et al., 2019). Our preliminary data also demonstrated that propidium iodide (PI)-positive necrotic RGCs increased drastically after 3 DIV (Supplementary Figure 1). Therefore, we collected retinae from the two-chamber culture system at 2 DIV.

**FIGURE 2:**
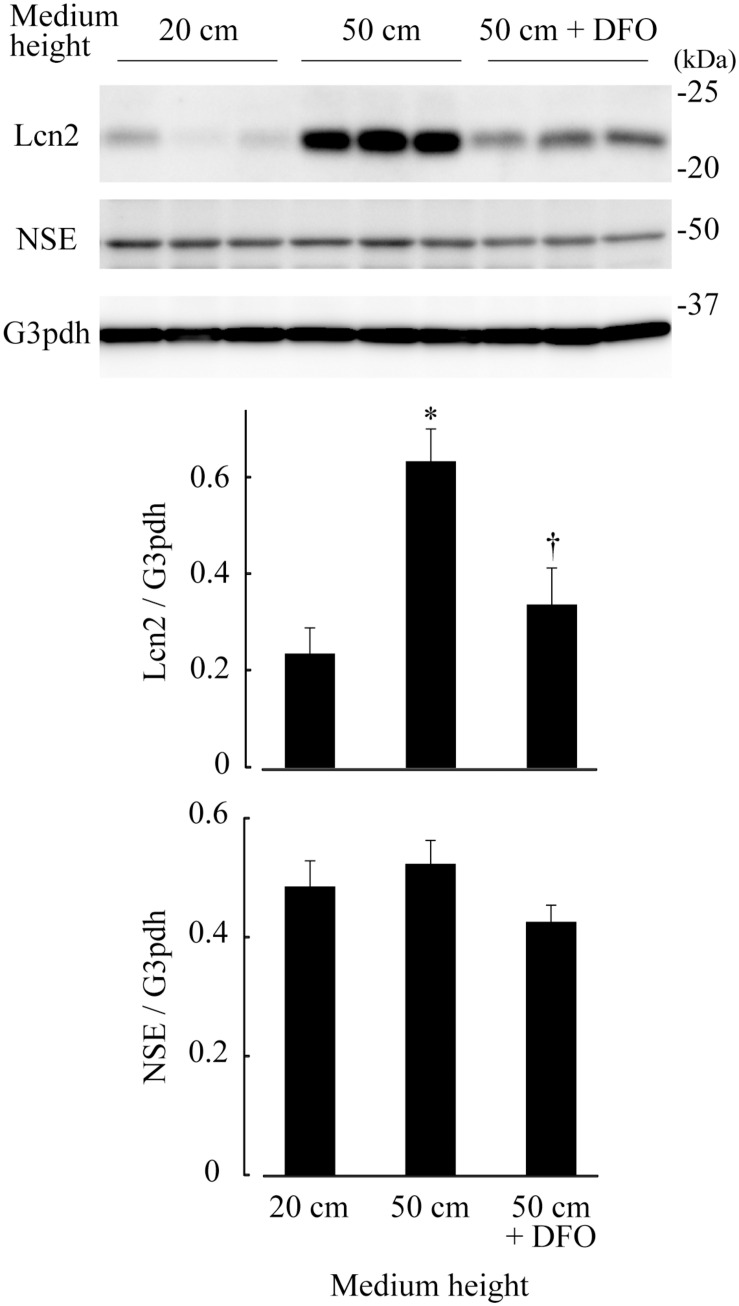
Lipocalin 2 (Lcn2) protein levels in retinae cultured under 20 cm H_2_O, 50 cm H_2_O, and 50 cm H_2_O with deferoxamine (DFO). C57BL/6J mouse retinae were cultured under medium heights of 20 cm and 50 cm, and 50 cm with 100 μM DFO. After 48 h, retinal tissue extracts were subjected to immunoblotting for Lcn2, neuron-specific enolase (NSE) and glyceraldehyde-3-phosphate dehydrogenase (G3pdh). Graphs represent relative protein levels of Lcn2 and NSE compared to the G3pdh loading control. *p ≤ 0.05 vs. 20 cm, ^†^*p* ≤ 0.05 vs. 50 cm.

Adult mouse retinae of C57BL/6J mice were subjected to pressure loads of 2 cm (atmospheric pressure), 20 cm (15 mm Hg, normal IOP), 50 cm H_2_O (37 mm Hg, high IOP), and then levels of the Lcn2 protein were examined by immunoblotting. After 48 h, Lcn2 protein levels were 2.68-fold higher in retinae cultured under 50 cm H_2_O pressure loads compared to those cultured under 20 cm H_2_O (*p* ≤ 0.01, Figure 2). The iron chelator deferoxamine was added to retinae cultured under 50 cm H_2_O pressure loads, which caused a 0.533-fold decrease in Lcn2 protein levels compared to the untreated 50 cm H_2_O pressure load control (*p* = 0.0270, Figure 2). No significant differences in the protein levels of NSE were detected in retinae among the pressure conditions, suggesting that the neuronal cell content in the retinal culture remained constant, regardless of medium height (Figure 2).

### RGC Apoptosis and Glial Activation Were Induced by Elevated Hydrostatic Pressure and Were Associated With Lcn2 Protein Levels

**FIGURE 3:**
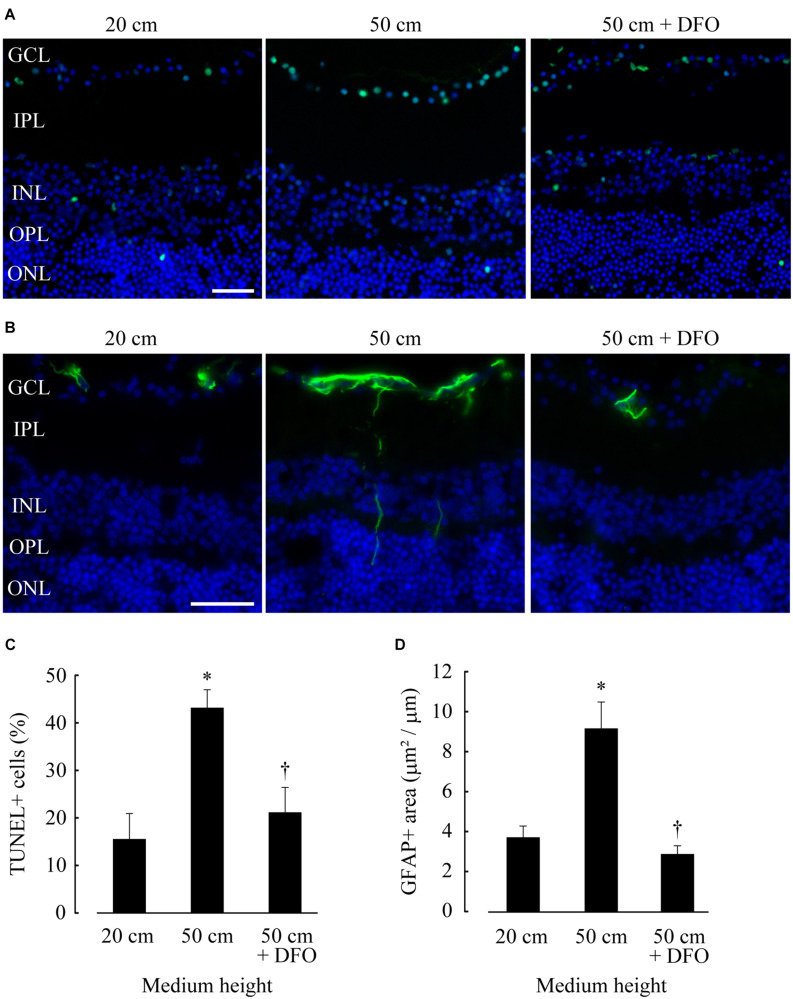
Retinal ganglion cell (RGC) apoptosis and glial activation increased at 50 cm H_2_O pressure and decreased following deferoxamine (DFO) treatment. **(A,B)** *In situ* TUNEL (A, green) and GFAP immunohistochemistry [**(B)**, green] assays were performed on frozen sections of retinae, and the sections were counterstained with DAPI [**(A,B)**, blue]. Bar = 40 μm. GCL, ganglion cell layer; IPL, inner plexiform layer; INL, inner nuclear layer; OPL, outer plexiform layer; ONL, outer nuclear layer. **(C,D)** The average percentage of TUNEL-positive nuclei in GCL (positive cell number/total cell number) and the average GFAP-positive area in retinae (positive area/horizontal length) are shown in **(C,D)**, respectively. *p ≤ 0.05 vs. 20 cm, ^†^*p* ≤ 0.05 vs. 50 cm.

We performed *in situ* TUNEL and GFAP immunohistochemical assays to quantify apoptotic cells and glial activation, respectively. TUNEL-positive cells were mostly found in the GCL, accounting for about 70 % of TUNEL-positive cells; a small population of TUNEL-positive cells were also found in the inner and outer nuclear layers (INL and ONL) (Figure 3A). The percentage of TUNEL-positive cells in GCL subjected to 50 cm H_2_O pressure loads (43.1%) was significantly higher compared to that under 20 cm H_2_O pressure (*p* = 0.0133). The percentage of TUNEL-positive cells significantly decreased following the addition of deferoxamine in 50 cm H_2_O pressure load cultures (*p* = 0.0274; 50 cm vs. 50 cm + deferoxamine; Figure 3C).

High GFAP immunoreactivity was present in the GCL, and GFAP-positive fibers were occasionally detected in the deeper layers of the retinae, indicating glial endfeet migration (Figure 3B); these results were comparable to those of previous study, in which the effects of hydrostatic pressure loading on explant retinal cultures were investigated (Ishikawa et al., 2010). Consistent with the results of the TUNEL assay, the GFAP-positive area was 2.46-fold higher in retinae cultured under 50 cm H_2_O pressure loads compared to that cultured under 20 cm H_2_O (*p* = 0.0285). The GFAP-positive area was comparatively lower in retinae supplemented with deferoxamine (*p* ≤ 0.01; 50 cm vs. 50 cm + deferoxamine; Figure 3D).

**FIGURE 4:**
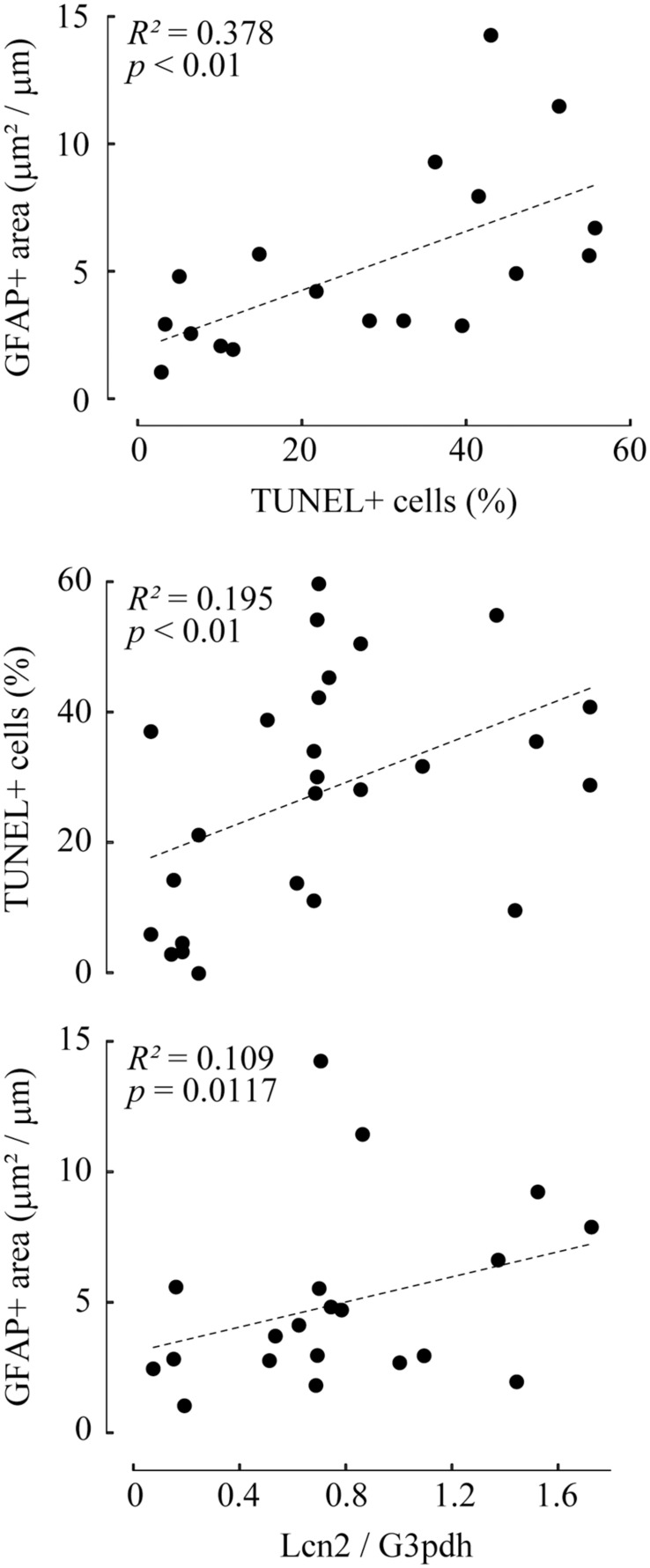
Correlation analyses between TUNEL-positive cells, GFAP-positive area, and lipocalin 2 (Lcn2) protein level. The values of TUNEL-positive cells and GFAP-positive area are represented as a scatter plot (top). The values of Lcn2 protein levels with TUNEL-positive cells (middle) or GFAP-positive area (bottom) are shown in the scatter plot. In each graph, the dot distribution approximates a linear function (dotted lines). Correlations and statistical significance were analyzed using Spearman’s rank test. *R*^2^- and *p*-values are shown.

Correlation analysis revealed that TUNEL-positive cells and GFAP-positive area were highly correlated (*R*^2^ = 0.378; *p* ≤ 0.01). Lcn2 protein levels were positively correlated with both TUNEL-positive cells (*R*^2^ = 0.195; *p* ≤ 0.01) and GFAP-positive area (*R*^2^ = 0.109; *p* = 0.0117), indicating that pressure-induced Lcn2 upregulation was linked to RGC apoptosis and glial activation (Figure 4). There were no significant differences between Lcn2 protein levels and RGC viability in retinae cultured under 2 cm and 20 cm H_2_O pressure loads, suggesting that low IOP may not directly affect the retina (Supplementary Figure 2).

### Extracellular Administration of Lcn2 Increased Apoptotic Cells in GCL; Deferoxamine Treatment Was Not Effective in Blocking Uptake of Recombinant Lcn2 in Retina

**FIGURE 5:**
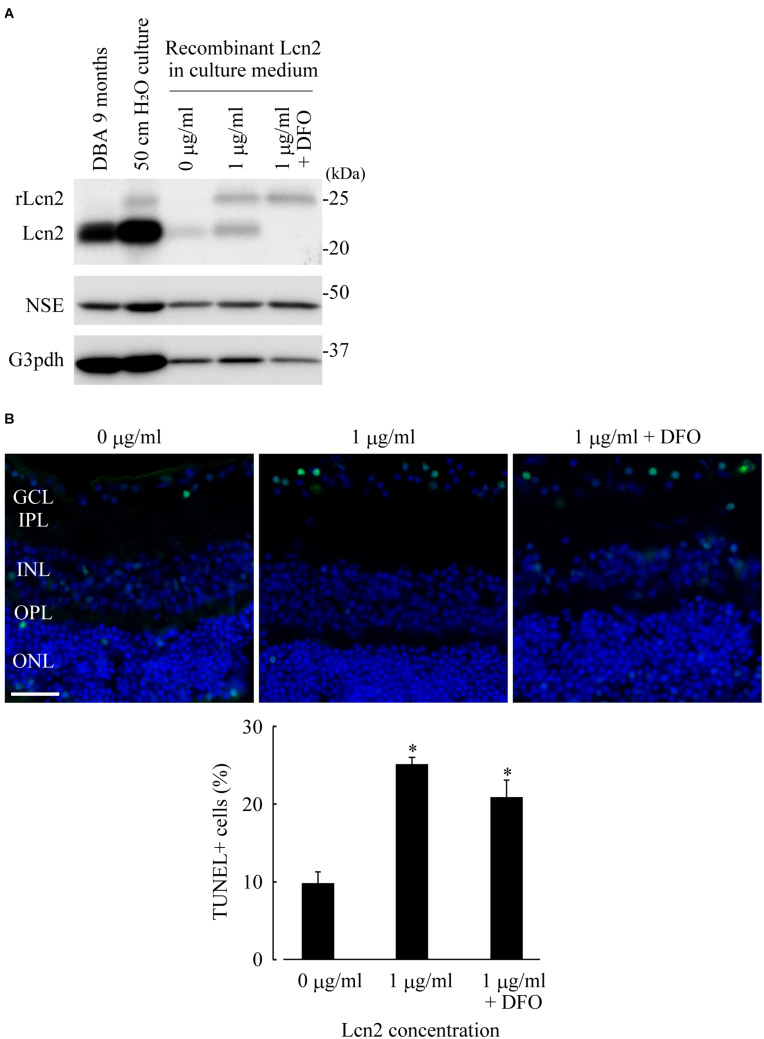
Lipocalin2 (Lcn2)-induced neurotoxicity in RGCs. C57BL/6J mouse retinae were cultured in the culture medium containing 1 μg/ml of recombinant mouse Lcn2 with or without 100 μM deferoxamine (DFO). After 16 h of culture, the retinae were subjected to immunoblotting analysis **(A)** or TUNEL assay **(B)**. **(A)** Lcn2 protein levels in the retina of DBA/2J mouse at 9 months of age, the cultured retina under 50 cm pressure, and the cultured retinae under atmospheric pressure with indicated treatment (0 μg/ml of recombinant Lcn2, 1 μg/ml of recombinant Lcn2, or 1 μg/ml of recombinant Lcn2 with 100 μM deferoxamine) were estimated by immunoblotting. Recombinant Lcn2 protein (rLcn2) appeared larger than native protein in immunoblotting, due to a polyhistidine-tag. **(B)** TUNEL assay (green) was performed on frozen sections of cultured retinae under atmospheric pressure with indicated treatment. The sections were counterstained with DAPI (blue). Bar = 40 μm. Graph represents the average percentage of TUNEL-positive cells out of total cells in GCL. *p ≤ 0.05 vs. 0 μg/ml.

Lcn2 neurotoxicity was assessed by addition of recombinant mouse Lcn2 protein to the culture medium of organotypic retinal explant cultures at atmospheric pressure. Significant reductions of cell number in GCL were observed when the retinae were treated with Lcn2 at doses higher than 1 μg/ml (Supplementary Figure 3). Immunoblotting analysis showed that recombinant Lcn2 protein was absorbed into retina cultured at dose of 1 μg/ml, which somehow leads to the increase of endogenous Lcn2 expression (Figure 5A); deferoxamine treatment did not change the protein level of absorbed recombinant Lcn2, but it decreased the protein level of endogenous Lcn2 (Figure 5A). After incubation of retinae in the presence of 1 μg/ml Lcn2 with or without deferoxamine, apoptotic cells in GCL were quantified using TUNEL assay. The percentage of TUNEL-positive cells in the presence of 1 μg/ml Lcn2 was 2.56-fold higher compared to the untreated control (*p* ≤ 0.01 vs. control; Figure 5B); deferoxamine treatment failed to decrease apoptotic cells in retina cultured with 1 μg/ml recombinant Lcn2 protein (*p* ≤ 0.01 vs. control; Figure 5B), possibly reflecting the protein level of absorbed recombinant Lcn2 in retina (Figure 5A). Overall, these data suggest that deferoxamine treatment attenuated pressure-induced retinal degeneration by inhibiting endogenous increase of Lcn2, but not by inhibiting retinal uptake of extracellular Lcn2.

### Upregulation of Lcn2 and Glial Activation Were Confirmed in the Retinae of DBA/2J Mice Exhibiting High IOP

**FIGURE 6:**
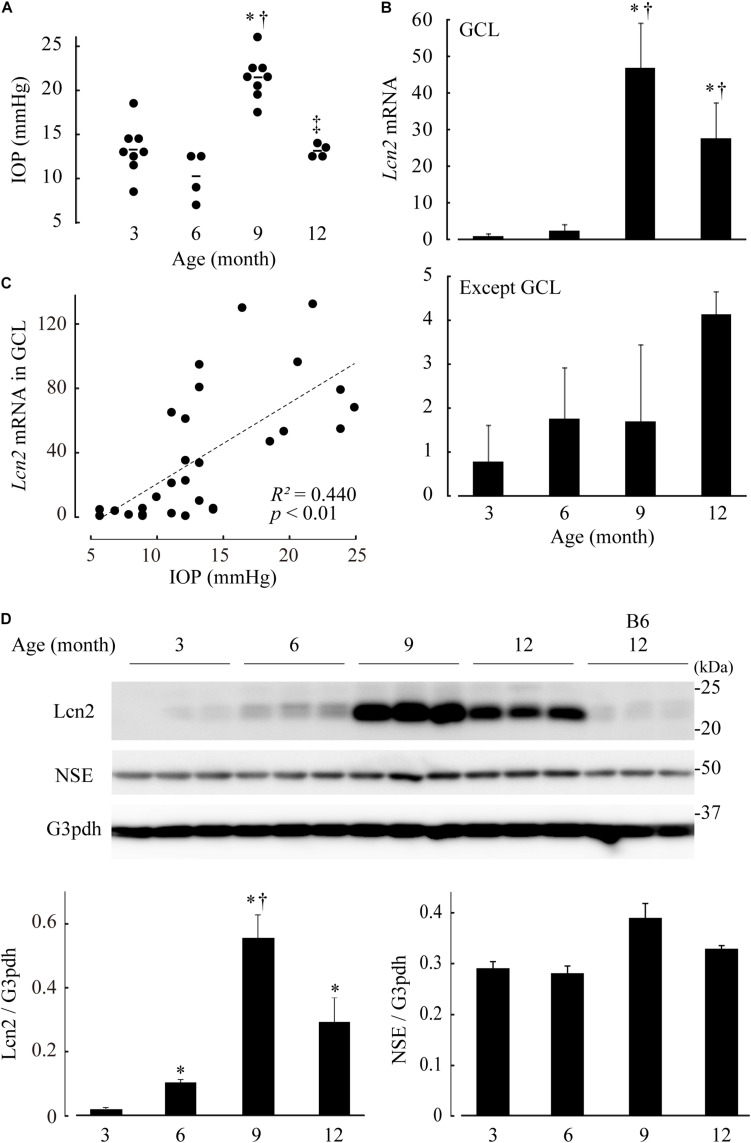
Elevated intraocular pressure (IOP)-induced lipocalin 2 (Lcn2) upregulation in retinae of DBA/2J mice. **(A)** IOP of DBA/2J mice at 3, 6, 9, and 12 months of age. **(B)** mRNA levels of *Lcn2* in dissected ganglion cell layer (GCL) (upper) and residual retinal layers (lower). Expression levels are represented as the mean ± standard error of fold changes compared to the level of the GCL at 3 months. **(C)** Correlation analyses between IOP and *Lcn2* mRNA levels. In each graph, the dot distribution approximates a linear function (dotted lines). Correlations and statistical significance were performed using Spearman’s rank test. A straight line of best fit (dotted lines), *R*^2^- and *p*-values are shown. **(D)** Lcn2 protein levels in the retinae of DBA/2J mice. Retinal tissue extracts of DBA/2J mice at each age were subjected to immunoblotting for Lcn2, neuron-specific enolase (NSE) and glyceraldehyde-3-phosphate dehydrogenase (G3pdh). Retinae of C57BL/6J (B6) mice were used as an aging control. Graphs represent relative protein levels of Lcn2 and NSE compared to the G3pdh loading control. *p ≤ 0.05 vs. 3 months, ^†^*p* ≤ 0.05 vs. 6 months, ^‡^*p* ≤ 0.05 vs. 9 months.

DBA/2J mice exhibit elevated IOP and a loss of RGCs, and are therefore used as models for glaucoma. To confirm our *in vitro* results, we monitored Lcn2 expression in the retinae of DBA/2J mouse at 3, 6, 9, and 12 months of age. *Lcn2* mRNA levels in the GCL were highest at 9 months of age, the same age at which IOP elevation peaks (Figures 6A,B). *Lcn2* expression levels in the GCL correlated positively with IOP (Figure 6C). At 9 months of age, *Lcn2* mRNA levels were 27.7-fold higher in the GCL than in the outer layers, indicating that *Lcn2* was expressed mainly by the GCL cells (Figure 6B). The upregulation of the Lcn2 protein in DBA/2J mouse retina was confirmed by immunoblotting; Lcn2 upregulation in retinal tissues occurred after 6 months, peaked at 9 months, and remained high after 12 months of age [*p* = 0.0206 (6 months), *p* = 0.0206 (9 months), *p* = 0.0329 (12 months) vs. 3 months; *p* = 0.0206, 6 vs. 9 months; Figure 6D]. This progressive increase in Lcn2 protein was not observed in age-matched C57BL/6J mice. Immunohistochemical analyses of DBA/2J retinae at 9 months of age exhibited Lcn2 protein localization to the GCL (Supplementary Figure 4), indicating that the Lcn2 protein was produced in and localized to the GCL. Levels of the NSE protein, which is primarily expressed in the GCL, inner to outer plexiform layers, did not change with age in DBA/2J mice (Figure 6D and Supplementary Figure 4).

**FIGURE 7:**
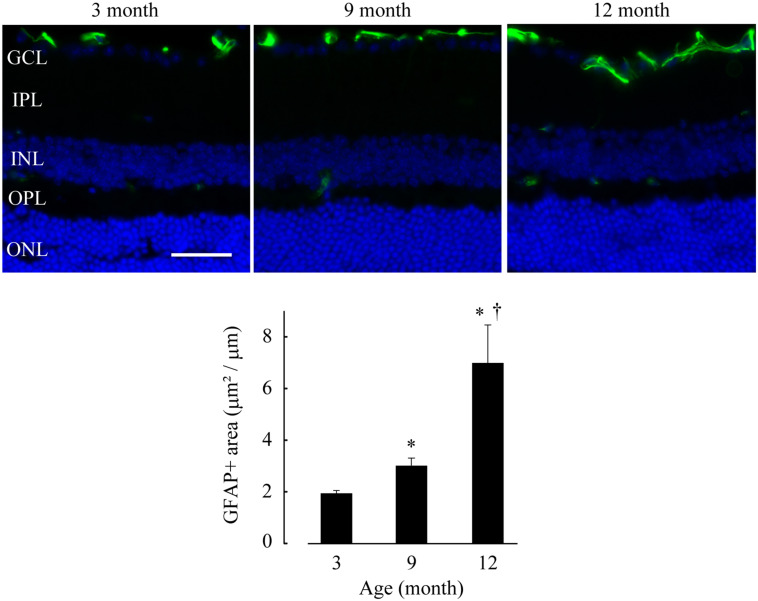
Glial activation was observed in the retinae of DBA/2J mice after 9 months of age. GFAP immunohistochemistry (green) was performed on frozen sections of DBA/2J mice retinae at 3, 9 and 12 months of age. The sections were counterstained with DAPI (blue). Bar = 40 μm. The average GFAP-positive area in retinae (positive area/horizontal length) is shown in a graph. *p ≤ 0.05 vs. 3 months, ^†^*p* ≤ 0.05 vs. 9 months.

We also examined the retinae of DBA/2J mice at 3, 9, and 12 months using a GFAP immunohistochemical assay and found a significant age-dependent increase in GFAP-positive area [*p* = 0.0178 (9 months), *p* = 0.0110 (12 months) vs. 3 months; *p* = 0.0178, 9 vs. 12 months], revealing that progressive gliosis had occurred as a result of IOP elevation (Figure 7). This feature is well consistent with the previous studies that RGC loss has become apparent after 8–10 months of age and progressed afterward in DBA/2J mice (Libby et al., 2005; Schlamp et al., 2006). Again, neither Lcn2 increase in the GCL nor glial activation were detected in age-matched C57BL/6J mice (Supplementary Figure 5).

## Discussion

Using our two-chamber culture system, we discovered that RGC apoptosis and glial activation were induced under 50 cm H_2_O pressure loads, and that the upregulation of Lcn2 correlated with both changes. Lcn2 upregulation and glial activation were confirmed in DBA/2J mice, and comparable Lcn2 protein levels were detected *in vitro* in the pressure-exposed retinae. Compared to existing devices, in which pressure chambers are controlled by gas flow, our pressure loading system offers some advantages. Our system enables modest pressure loads (≤ 37 mm Hg), which is similar to the pressure range observed during human ocular hypertension (24–32 mm Hg, Kass et al., 2002); however, most studies that used artificial pressure chambers worked within a pressure range of 40–80 mm Hg (Tezel and Wax, 2000; Sappington et al., 2009; Ishikawa et al., 2010, 2016; Osborne et al., 2015; Liu et al., 2017, 2019; Fischer et al., 2019). Since our system does not require any specialized equipment, it can be installed in a common CO_2_ incubator and would be compatible with a wide variety of culture types (Hagiyama et al., 2017; Yoneshige et al., 2017).

To our knowledge, this is the first report demonstrating the upregulation of Lcn2 in direct response to elevated hydrostatic pressure in an experimental retinal model. Our data suggest that Lcn2 is produced and functions in the GCL of the retina. However, the mechanisms by which cells sense changes in pressure and produce Lcn2 remain unclear. To date, investigations into the molecular mechanisms behind pressure sensing in the retina are limited. Sappington et al. found that RGCs underwent apoptosis at 70 mm Hg pressure and that this was mitigated by transient receptor potential vanilloid 1 (TRPV1) antagonism or extracellular Ca^2+^ chelation. This suggested that TRPV1 was activated by elevated hydrostatic pressure, which increased intracellular Ca^2+^ levels and led to RGC apoptosis (Sappington et al., 2009). TRPV4 is involved in a pressure-triggered mechano-transduction process similar to TRPV1 (Ryskamp et al., 2011). Choi et al. showed that astrocytes in the optic nerve head express a variety of mechanosensitive channel proteins, such as the transient receptor potential (TRP) channels, Piezo1 and Piezo2 (Choi et al., 2015). Furthermore, Piezo agonists and antagonists can suppress and promote neurite outgrowth in RGCs, respectively (Morozumi et al., 2020). Beckel et al. (2014) found that mechanical stretching stimulates ATP release from optic nerve head astrocytes and that pannexin channels may mediate this process.

Astrocytes are thought to be a primary source of Lcn2 in the brain. The expression of Lcn2 in primary astrocytes increases following exposure to cytotoxic agents such as lipopolysaccharide or tumor necrosis factor α (Lee et al., 2009; Gasterich et al., 2020), Recombinant Lcn2 protein induces GFAP upregulation and morphological changes in primary astrocytes, suggesting that Lcn2 performs an autocrine function in astrocytes (Lee et al., 2009; Jang et al., 2013). Studies in Lcn2 knockout mice have shown that neurodegenerative phenotypes, including gliosis in diabetic neuropathy (Bhusal et al., 2020) and encephalopathy (Bhusal et al., 2019), intracerebral hemorrhage (Ni et al., 2015; Mao et al., 2016; Zhang et al., 2021), cerebral ischemia (Jin et al., 2014; Kim et al., 2017; Zhao et al., 2019), spinal cord injury (Rathore et al., 2011), and optic neuritis (Chun et al., 2015), are attenuated in Lcn2 knockouts. Chun et al. showed that Lcn2 expression increases in the optic nerve astrocytes of experimental autoimmune optic neuritis (EAON) model and that optic neuritis features such as demyelination and gliosis are reduced in EAON-induced Lcn2 knockout mice (Chun et al., 2015). It is speculated that Lcn2 knockout mice are also resistant to IOP elevation-induced glaucomatous changes in respect to gliosis. Interestingly, Lcn2 expression in astrocytes is dependent on the activation of the transcription factor signal transducer and activator of transcription 3 (STAT3), and this pathway is mediated by the transient receptor potential canonical (TRPC) channel (Shiratori-Hayashi et al., 2015, 2020). We speculate that retinal glial cells, such as Müller cells and astrocytes, may sense pressure increases via TRPC-mediated Ca^2+^ signals, which then trigger Lcn2 synthesis. Furthermore, glial Lcn2 may function as an autocrine and paracrine mediator of glial cell proliferation and RGCs apoptosis, respectively. By taking advantages of the high versatility of our culture system, additional studies using isolated neurons or glia of retina cultured under increased pressure conditions should be performed, in combination with a pharmacological agonist or antagonist of mechanosensitive channels (Schnichels et al., 2020).

Iron-chelating agents can improve the clinical symptoms of Alzheimer’s disease (Crapper McLachlan et al., 1991; Prasanthi et al., 2012) and Parkinson’s disease (Devos et al., 2014; Carboni et al., 2017). Lcn2 is an iron-binding protein (Devireddy et al., 2005); therefore, we hypothesized that iron chelators may attenuate Lcn2 function. Although we found that the iron chelator deferoxamine is neuroprotective against pressure-induced retinal degeneration, the precise molecular mechanism behind the protective effects of deferoxamine remains unclear. Consistent with our findings, deferoxamine treatment reduces Lcn2 revels in injured rat brains (Dong et al., 2013; Zhao et al., 2016), and Lcn2-induced neurotoxicity in dopaminergic neurons of the substantia nigra is decreased following deferoxamine treatment in mice (Kim et al., 2016). Amyloid beta promotes iron accumulation in astrocytes, and amyloid beta-induced Lcn2 production is inhibited by deferoxamine treatment (Dekens et al., 2020). Moreover, oral administration of the iron chelator deferiprone protects against RGC and optic nerve loss in glaucoma mouse models with ocular hypertension induced by microbead injections (Cui et al., 2020). Our preliminary study investigating the effect of iron overload on organotypic retinal explant cultures by using cell-permeable ferric 8-hydorxyquinoline complex (Fe-8HQ) (Fang et al., 2018) showed that RGC loss became obvious when retinae were treated with Fe-8HQ at doses higher than 100 μM and that TUNEL-positive cells in the presence of 10 μM Fe-8HQ was 4.24-fold higher compared to the untreated control (*p* ≤ 0.01 vs. control; Supplementary Figure 6). Inconsistent with the observation in the pressure-treated retinae (Figure 3A) nor in the Lcn2-treated retinae (Figure 5B), TUNEL-positive cells were detected in all layers of retinae treated with Fe-8HQ (Supplementary Figure 6), suggesting that the neurotoxicity of iron overload was not specific to RGC. Whether iron dysregulation occurred within the pressure-treated retinae in our system and how this relates to Lcn2 upregulation should be determined in future studies, alongside investigations into the potential therapeutic application of iron chelators in the treatment of glaucoma.

## Data Availability Statement

The raw data supporting the conclusions of this article will be made available by the authors, without undue reservation.

## Ethics Statement

The animal study was reviewed and approved by the Committee for Animal Experiments of Kindai University (protocol code KAME-29-001).

## Author Contributions

AY and AI: conceptualization. AY and MH: methodology. MH, TI, and RK: validation. AY: formal Analysis, visualization, and writing—original draft preparation. AY, YT, and SU: investigation. MH and YK: resources. TI, YK, and AI: writing—review and editing. AI: supervision and project administration. AY, MH, and AI: funding acquisition. All authors contributed to the article and approved the submitted version.

## Conflict of Interest

The authors declare that the research was conducted in the absence of any commercial or financial relationships that could be construed as a potential conflict of interest.
