# Supplementary material for: Elevated Hydrostatic Pressure Causes Retinal Degeneration Through Upregulating Lipocalin-2
Source: Front Cell Dev Biol. 2021 May 31;9:664327. doi: 10.3389/fcell.2021.664327 (PMC8201777; doi:10.3389/fcell.2021.664327)
Supplement: Supplementary file 1 [file Data_Sheet_1.docx]

Supplementary Material

## Supplementary Figures and Legends


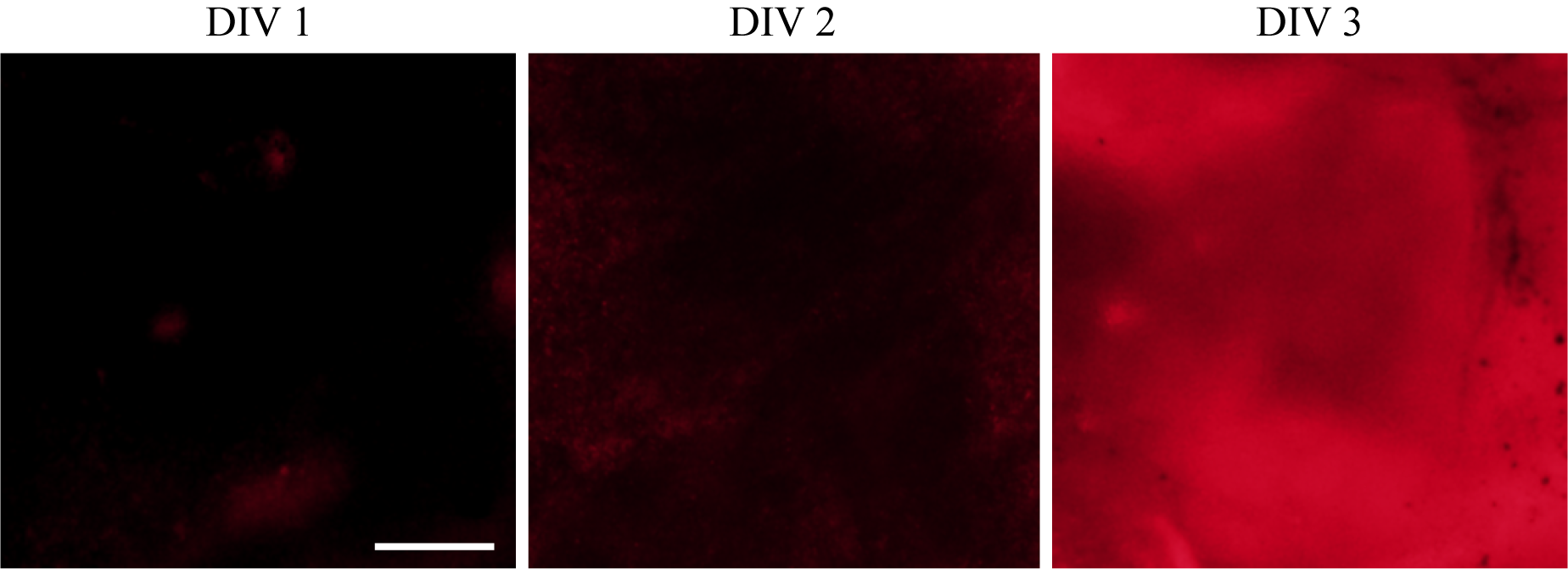


**Figure S1 Whole mount propidium iodide (PI) staining of organotypic retinal explant cultures at atmospheric pressure**

After 1, 2, and 3 days *in vitro* (DIV), the cultured retinae were incubated with 1 μg/ml of PI for 1 h and the vitreal surface of retina was mounted face up to visualize PI signals (red) from GCL under a fluorescent microscope. Bar = 250 µm.


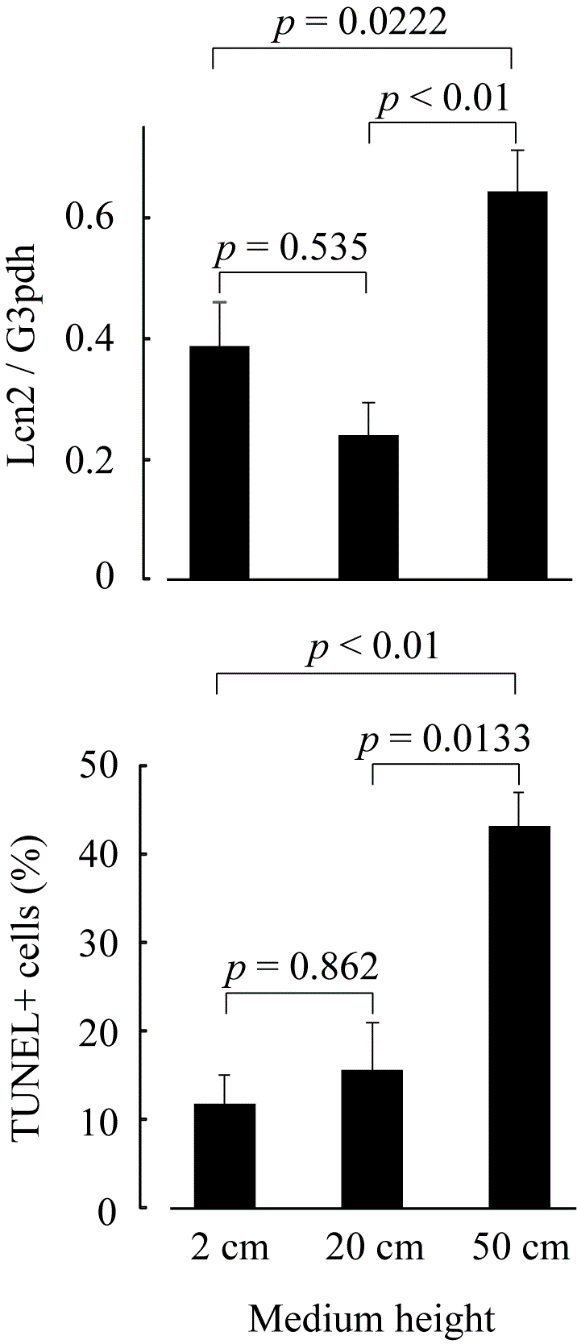


**Figure S2 Lipocalin 2 (Lcn2) protein levels and the percentage of TUNEL-positive cells in the ganglion cell layer (GCL) of retinae cultured at atmospheric pressure.**

Lcn2 protein levels (upper) and TUNEL-positive cells (lower) in the retinae at atmospheric pressure (2 cm H2O) were calculated as described in the main text and shown in the graphs.


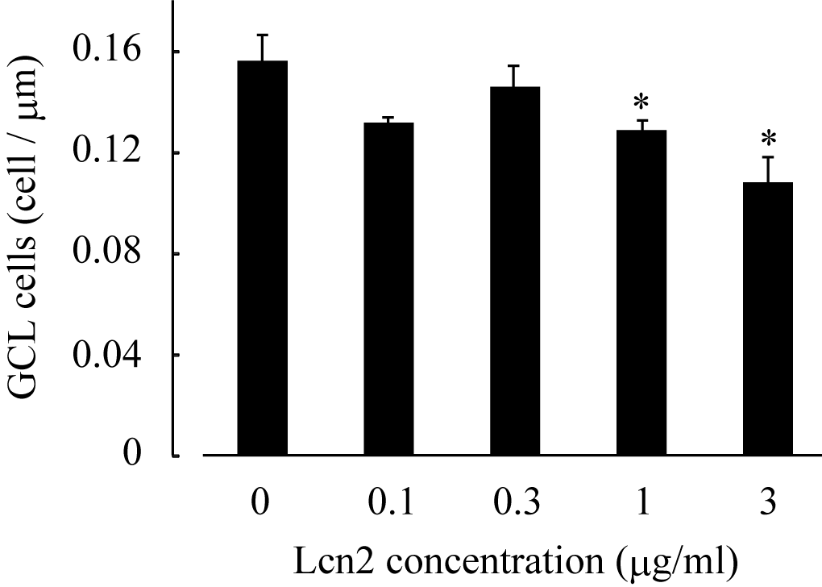


**Figure S3 GCL cell number in the retinae cultured with various concentrations of recombinant lipocalin 2 (Lcn2)**

Retinae were treated with five doses of recombinant mouse Lcn2 protein (0, 0.1, 0.3, 1, and 3 µg/ml) and GCL cells were counted. The average values of GCL cell number (cell number/horizontal length) are shown in graph. *: *p* ≤ 0.05 vs. 0 µg/ml.


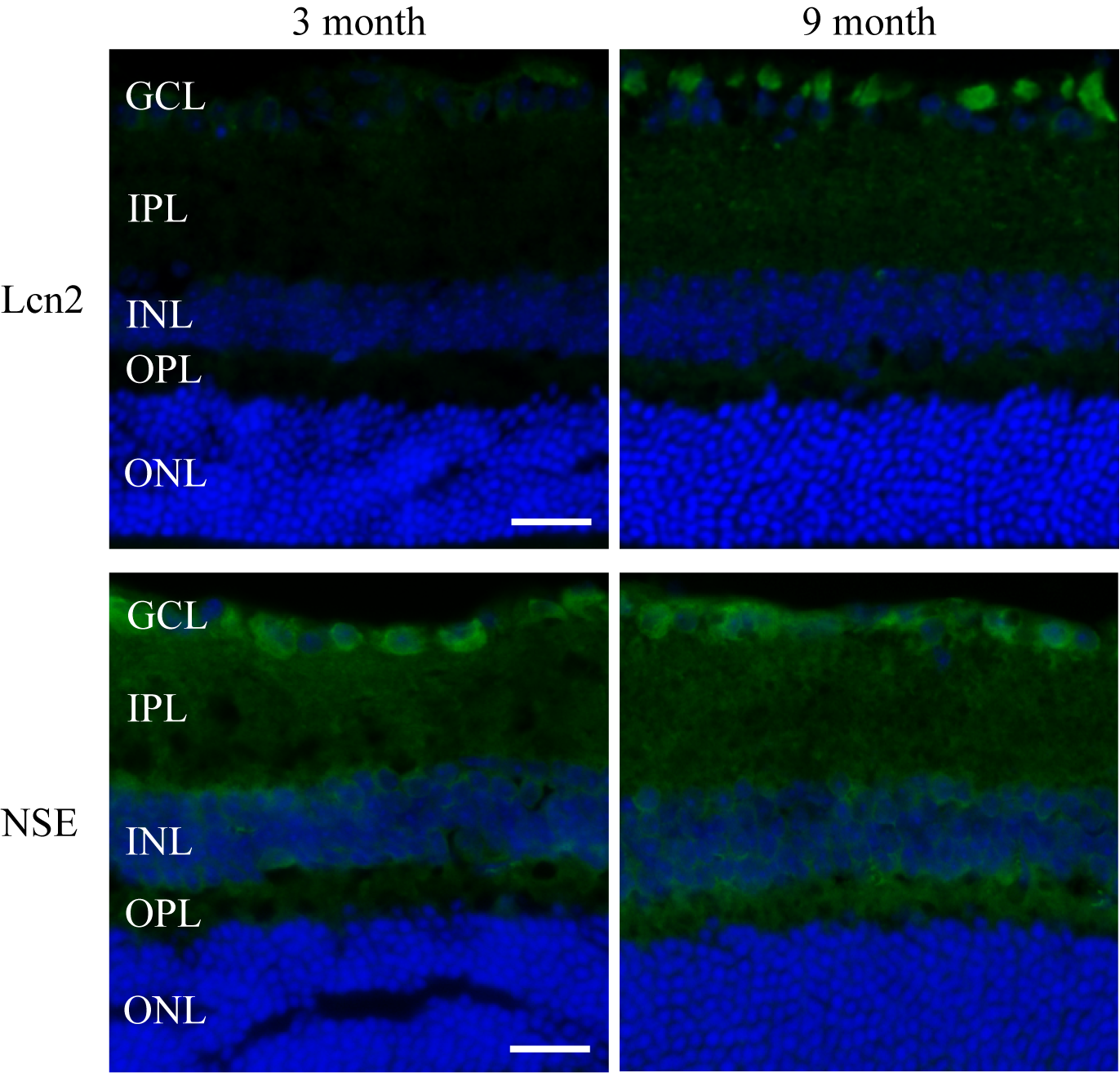


**Figure S4 *In situ* distributions of lipocalin 2 (Lcn2) and neuron-specific enolase (NSE) in the retinae of DBA/2J mice**

Lcn2 (upper) and NSE (lower) distributions in the retinae of DBA/2J mice at 3 or 9 months of age were shown in green and DAPI staining were shown in blue. Bar = 40 µm.

**
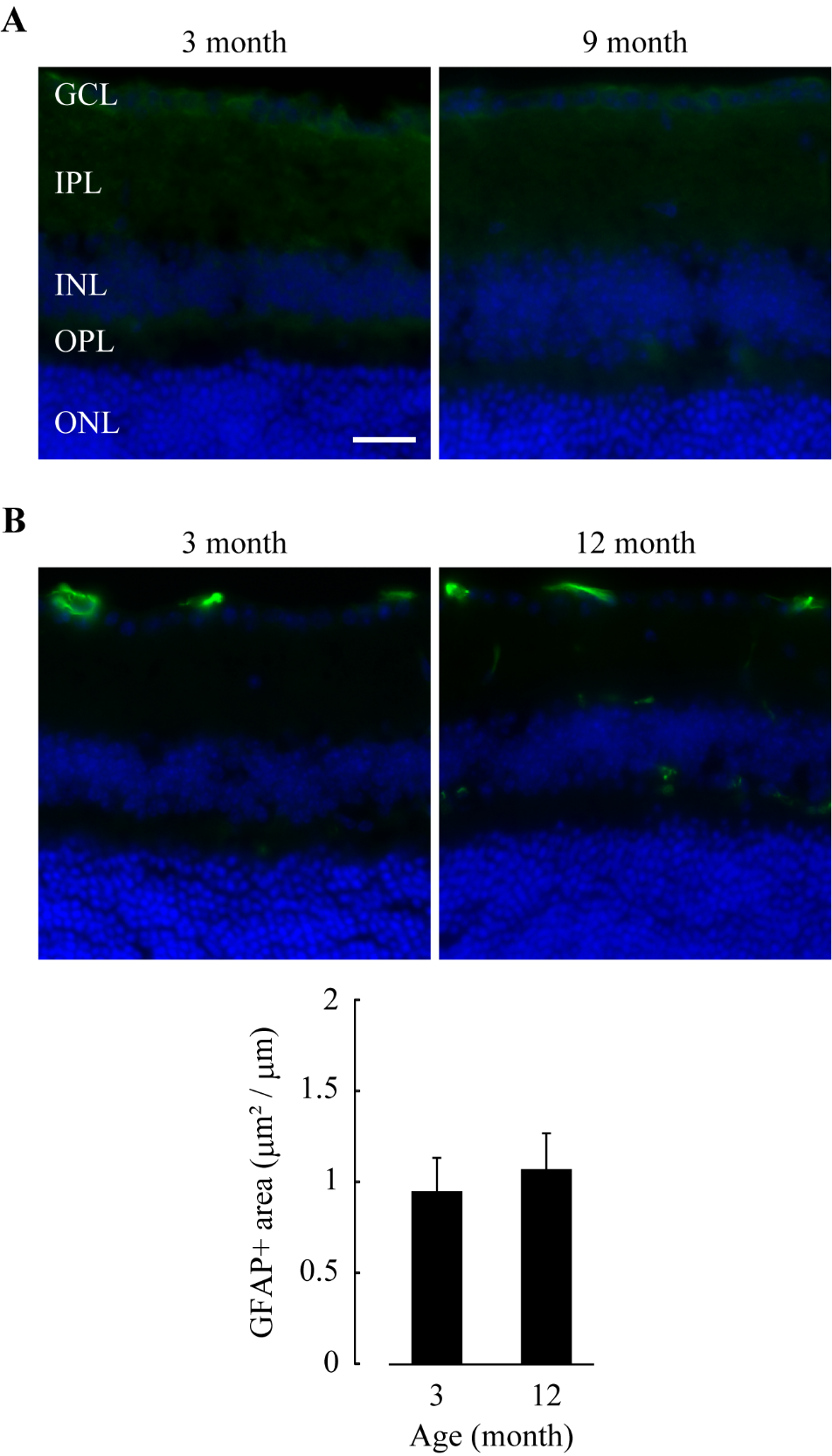
**

**Figure S5 *In situ* distribution of lipocalin 2 (Lcn2) and glial fibrillary acidic protein (GFAP) in the retinae of C57BL/6J mice**

(A, B) Lcn2 (A) and GFAP (B) distributions in the retinae of C57BL/6J mice at 3, 9, or 12 months of age were shown in green and DAPI staining were shown in blue. Bar = 40 µm. (B, graph) The average GFAP-positive area in retinae (positive area/horizontal length) are shown.

**
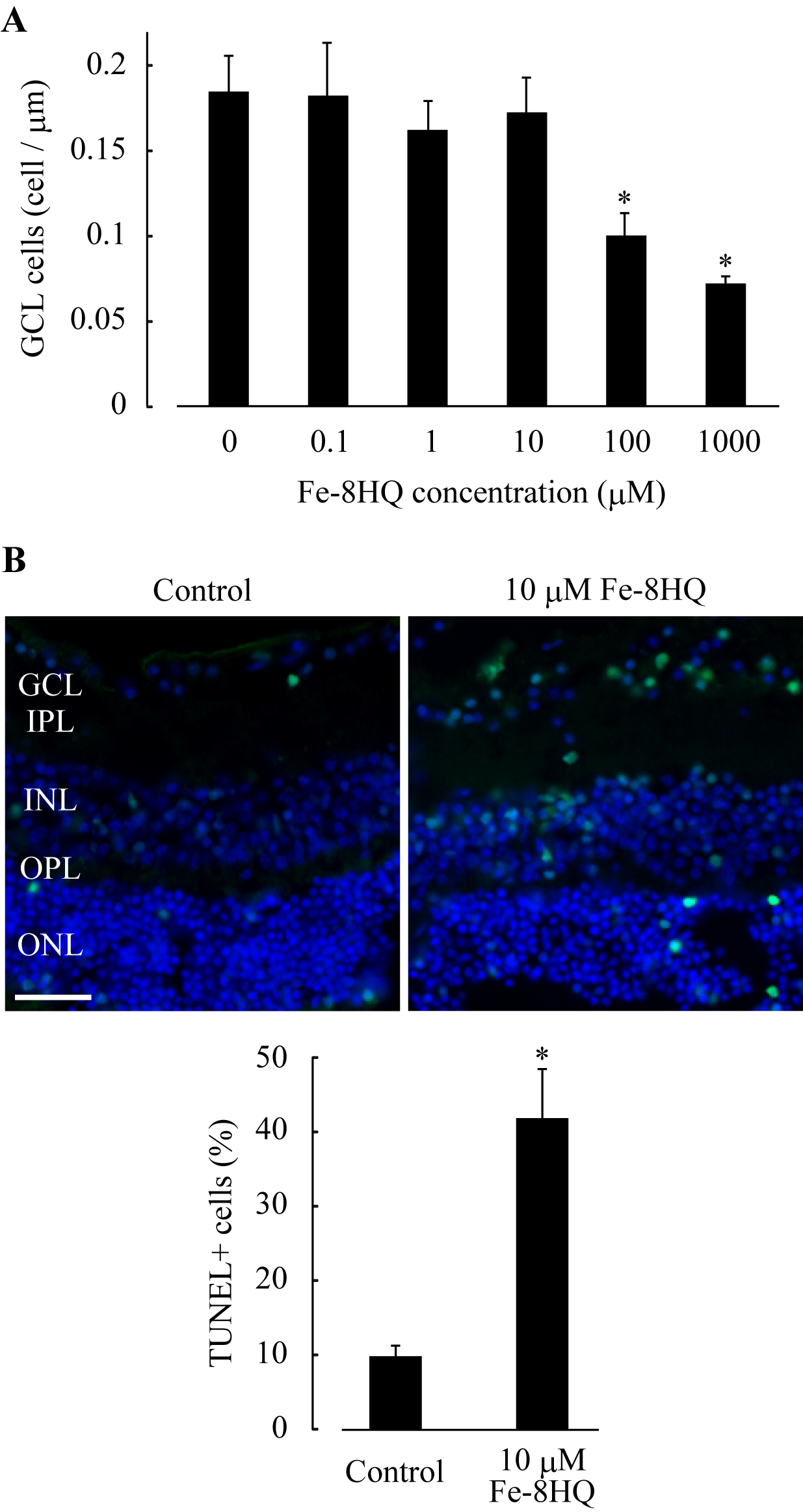
**

**Figure S6 Iron-induced neurotoxicity in retina**

(A) Retinae were treated with ferric 8-hydorxyquinoline complex (Fe-8HQ; 1:1; ferric chloride, Sigma-Aldrich, MO, USA; 8-hydroxyquinoline, Tokyo Chemical Industry Co., Ltd., Tokyo, Japan) at indicated doses and GCL cells were counted. The average values of GCL cell number (cell number/horizontal length) are shown. *: *p* ≤ 0.05 vs. 0 µM.

(B) C57BL/6J mouse retinae were cultured in the culture medium containing 10 µM of Fe-8HQ. After 16 h of culture, the retinae were subjected to TUNEL assay (green) and counterstaining with DAPI (blue). Bar = 40 µm. Graph represents the average percentage of TUNEL-positive cells out of total cells in GCL. The control retinae were identical to those in Figure 5B. *: *p* ≤ 0.05 vs. control.
